# MMPBSA Decomposition of the Binding Energy throughout a Molecular Dynamics Simulation of Amyloid-Beta (Aß_10−35_) Aggregation

**DOI:** 10.3390/molecules15042730

**Published:** 2010-04-15

**Authors:** Josep M. Campanera, Ramon Pouplana

**Affiliations:** Departament de Fisicoquímica, Facultat de Farmàcia, Universitat de Barcelona, Av. Joan XXIII, s/n, Diagonal Sud, 08028 Barcelona, Catalonia, Spain

**Keywords:** Alzheimer’s disease, amyloid-beta (Aβ) peptides, dimerization, replica exchange molecular dynamics (REMD), binding free energy calculations, Kepler scientific workflow

## Abstract

Recent experiments with amyloid-beta (Aβ) peptides indicate that the formation of toxic oligomers may be an important contribution to the onset of Alzheimer’s disease. The toxicity of Aβ oligomers depend on their structure, which is governed by assembly dynamics. However, a detailed knowledge of the structure of at the atomic level has not been achieved yet due to limitations of current experimental techniques. In this study, replica exchange molecular dynamics simulations are used to identify the expected diversity of dimer conformations of Aβ_10−35_ monomers. The most representative dimer conformation has been used to track the dimer formation process between both monomers. The process has been characterized by means of the evolution of the decomposition of the binding free energy, which provides an energetic profile of the interaction. Dimers undergo a process of reorganization driven basically by inter-chain hydrophobic and hydrophilic interactions and also solvation/desolvation processes.

## 1. Introduction

Alzheimer’s disease (AD) involves amyloid-β (Aβ) accumulation. The progressive accumulation of Aβ aggregates is widely believed to be fundamental to the initial development of neurodegenerative pathology and to trigger a cascade of events such as neurotoxicity, oxidative damage and inflammation that contribute to the progression of AD [1,2,3,4,5,6]. Recent studies on Aβ proteins support the idea that soluble oligomers are the pathogenic components that drive neurodegeneration and neuronal cell death, rather than mature amyloid fibrils [7]. Therefore, the inhibition and/or reversion of the early stages of Aβ oligomerization is an attractive therapeutic approach for targeting the underlying disease progression of AD. One of the main Aβ proteases, IDE, catabolizes natural Aβ monomers but no soluble dimers and trimers [8]. Considering the importance of soluble oligomeric Aβ forms in AD pathogenesis, it is now clear that drug development in this area should focus on inhibitors of the oligomerization of Aβ rather than inhibitors of fibril formation.

Aβ self-aggregation is driven by hydrophobic-hydrophobic interactions as well as the salt bridges and is accompanied by conformational transition from the non-pathogenic random coil to the pathogenic β-sheet form. Aβ fibrillization involves formation of dimers, trimers, tetramers and small oligomers, followed by growth into protofibrils and fibrils via a complex multistep-nucleated polymerization. The aggregation process from Aβ monomer peptides via soluble oligomeric states to fibrils is a complex dynamic event that depends critically on the peptide concentration, pH and solvent properties. Structural studies *in vitro* have shown that Aβ fibril formation is preceded by formation of intermediates, spherical oligomeric states, and protofibrils [9].

The Aβ plaques in AD brain are predominantly comprised of two Aβ alloforms: Aβ_1–40_ and Aβ_1–42_. Thus, the fibrillar structure of Aβ peptide aggregates is relatively well established. Experiments have targeted the structure of Aβ fibrils using electron microscopy, X-ray diffraction, electron paramagnetic resonance spectroscopy and solid-state NMR spectroscopy [10]. The amino acid sequence of Aβ_1–42_ is DAEFRHDSGYEVHHQKLVFFAEDVGSNKGAIIGLMVGGVVIA in which the boldface letters correspond to the 26 central residues, Aβ_10–35_. The solid-state NMR study by Zhang *et al.* [11] showed that the full length Aβ_1–40_ and Aβ_1–42_ retain the same structural features as Aβ_10–35_, indicating that Aβ_10–35_ can represent the full length of structural features very well. Moreover, it was found that both Aβ_10–35_ and Aβ_1–42_ have an in-register parallel β strand structure. It was also found that the Aβ_1–40_ has two β-strands connected with a turn region around 25–29 residues. According to this study, the 1–11 residues are unstructured and 12–24 and 30–40 have the β strand structure. A computational study of Aβ_16–35_ and Aβ_10–35_ at high temperature by Ma and Nussivov [12] supports the same feature. It is noted that the two β*-*strand regions have hydrophobic residues (LVFFA, 17–21 residues, and GAIIGLM, 29–35 residues) and the bend region has polar and charged residues. In the previous amino acid sequence, the hydrophobic LVFFA core is underlined. Additionally, Tjernberg *et al.* [13] reported that the central region (amino acid residues 16–20) of Aβ monomers is responsible for its self-association and aggregation. Furthermore, previous studies suggest that amino acid residues within or close to Aβ_16-20_ are important for the adoption of the correct β-pleated sheet structure of Aβ and the proteolytic processing of its precursor. Here, it is also shown that this region harbors a binding sequence required for the polymerization of Aβ into soluble oligomeric states. Kirkidatze *et al.* [14] demonstrated that α-helix formation is a key step in Aβ fibril assembly and revealed that the substitution of Asp23 by Asn or Lys retards fibrillogenesis. This data emphasize the importance of Asp23 in controlling Aβ holding and assembly. Early studies of fibril formation by Aβ peptide fragments terminating at Lys28 [15] have suggested that Asp23 and His13 may function in the formation of salt-bridges. In addition, these studies suggested that His13 might be involved in intersheet packing interactions.

With regard to the energetic profile of the interaction between both monomers, there exist two views. On the one hand, some researchers state that the interaction is predominantly hydrophobic. This hypothesis is based on the idea that the major driving force for the oligomerization of Aβ_10-35_, which contains the crucial central hydrophobic cluster essentials, is the hydrophobic interaction between these central hydrophobic regions whereas the hydrophilic residues are primarily stabilized by interstrand backbone-backbone and side chain-side chain hydrogen bonds (Glu22 and Asp23) [16,17]. However, Kirkidatze *et al.* [14] have suggested that the “hydrophobic collapse” may be caused by the presence of the uncharged Glu22 and Asp23 residues at low pH and consequently it would produce a decrease in electrostatic potential in the central region and cause an enhancement of interactions between uncharged side-chains. On the other hand, research studies also point out the importance of the hydrophilic contributions in the interchain area exemplified by high density of inter hydrogen bonds and conceptualized under the idea of the generic principle of amyloid self-assembly (PASA) [18,19]. These experimental studies emphasizes that the favorable electrostatic interactions must play a role in the early structural organization of soluble Aβ oligomers. For instance, Sato *et al.* [20] on the basis of solid-state NMR measurements, presents structures of Aβ fibrils that implicate the folding of the N-terminal region back onto the C-terminus which incorporate the hydrophilic interaction between Phe4, His6 or Tyr10 against Gly33 at early stages in fibril formation.

Despite the limitations of conventional experiments in the study of the amyloid-β structures, mainly due to its poor solubility and difficulty of forming single crystals, the main structural features have begun to appear using *in-silico* approaches. In the present computational work, we first studied the self-assembly of Aβ_10–35_ dimer formation at the atomic level using extensive replica exchange molecular dynamics (REMD) simulations. Secondly we characterized the hydrophobic-hydrophobic and electrostatic interactions in the most representative dimer conformation as well as the effect of solvation on the aggregation by means of the decomposition of the binding free energy using MMPBSA procedure. It is the first time, as far as we know, that an accurate decomposition of the binding free energy such as MMPBSA is applied to this peptide. Finally, from the methodological point of view, our efforts are in the direction to combine all analyses in order to configure a unique view of the dynamic process. Since diverse and unconnected software is needed to perform such analyses we introduced Kepler scientific workflow system to orchestrate all analyses under the same software environment.

## 2. Computational Methods

### 2.1. Molecular dynamics simulation on a single monomer

Initially, a molecular dynamics simulation for a single monomer of Aβ_10–35_ with YEVHHQKLVFFAEDVGSNKGAIIGLM amino acid sequence was performed in order to get an initial monomer structure to build up the dimer. The initial conformation of Aβ_10–35_ strand was obtained from the NMR structure (PDB code 1HZ3) [11], which adopts a collapsed coil structure in water. The Amber99 force field was used as implemented in the AMBER9 package [21]. The standard protonation state at physiological pH (7.4) was assigned to the ionizable residues. It is important to fix the pH since Kirkidatze showed that the most rapid kinetics occurs in the pH range 4–4.5 and that the kinetics exhibits two sharp transitional zones which were coincident with the pKa values of Asp and His [14]. In the next step the monomer structure was solvated with TIP3P waters in an octahedral box. Periodic boundary conditions and Ewald sums (grid spacing of 1 Å) were used to treat long range electrostatic interactions [22]. The nonbonded cut off distance was maintained at 15 Å and the temperature and pressure was controlled by Berendsen thermostat and barostat with coupling constant of 0.1 ps.

The equilibration steps that allow both monomer and water to relax to a local minimum are as follows: (1) minimize the energy of the water while peptide is kept immobile, (2) perform molecular dynamics (MD) simulation on the water using the NVT ensemble at a range temperature of 0–200 K for 150 ps (the time step is 1 fs) while peptide are kept immobile, (3) minimize again the energy of the water while peptide are kept immobile, (4) minimize the of energy of peptide while water molecules are kept immobile, (5) perform MD simulations of the peptide using the NVT ensemble at a range temperature of 0–100 K while water molecules are kept immobile, (6) minimize the energy of the peptide a second time while water molecules are kept immobile, (7) minimize the energy of the peptide and water molecules simultaneously and finally the production step, (8) perform the production run, *i.e.*, unconstrained molecular dynamics simulations on the peptide and water using the NPT ensemble that heats up to a temperature of 280 K and P = 1 bar for 300 ps. At steps 1, 3, 4, 6, and 7 we used the steepest descent energy minimization method. During steps 2 and 5, which are parts of equilibration, peptide and water coordinates have to reach a local energy minimum for the given force field and with respect to each other. The temperatures are kept so low that there are no conformational changes.

### 2.2. Replica exchange molecular dynamics (REMD) simulations of the dimer at 280–405 K

The initial conformation of the Aβ_10–35_ dimer for the MD simulation was obtained from two copies of the final structure of the MD simulation of the monomer in the previous section. The distance between each copy was initially about 20 Å. Considering that the end-to-end distance of Aβ_10–35_ is about 27 Å, this separation provides sufficient space for the overall tumbling of each Aβ_10–35_ monomer.

Firstly, a set of equilibration MD simulations for the dimer complex at NPT ensemble at 1 bar from 280 K to 405 K at 5 K/10 ps steps was performed. The MD simulation was equilibrated during 50 ps in each of the 26 temperatures. Secondly a final set of MD simulations at NPT ensemble at 1 bar during 6 ns for each of the 26 final structures obtained in the previous step was carried out. It was observed that the motion of each unit of Aβ_10–35_ was not hindered inside the simulation box during the initial stage of the simulation. It is important to notice that the simulation results are independent of the initial placement of Aβ_10–35_.

Finally, in order to explore the diversity of conformations adopted by the dimer, 26 MD simulations in the temperature range of 280–405 K were performed by means of replica exchange molecular dynamics (REMD) [23]. The MD simulation of each replica was undertaken at NPT ensemble at 1 bar during 19 ns. The initial conformation at each temperature was obtained from the final conformation of the equilibration MD explained above. It is noted that 405 K is high enough to make the Aβ oligomers into completely unstructured and dissociated conformation. The MD details that are not mentioned in this section are the same that those explained in the previous section.

### 2.3. Characterization of the dimer conformations and selection of the representative MD simulation

We observed the spontaneous self-assembly of oligomers of Aβ_10–35_ peptides during the time scale of our REMD simulations. The geometric analysis of the average structure of the final 2 ns in each of the 26 replicas shows that they can be grouped into the 10 possible conformation types predicted by Urbanc *et al*. for the Aβ_1–42_ peptide model [24]. Urbanc *et al.* based the geometric classification on the fact that at least one of the two monomers in the dimer complex is in a β-hairpin conformation with two strands largely distorted from the initial collapsed coil conformation. They named six of the ten conformations as NN-parallel, NC-parallel, CC-parallel, NN-antiparallel, NC-antiparallel and CC-antiparallel according to the inner two strands of the dimer (each strand is closer to either the N terminus or the C terminus) and the two inner strands are either parallel or antiparallel. The four rest conformations were termed as nested-parallel, nested-antiparallel, antinested-parallel, and antinested-antiparallel since only the inner monomer adopts a β-harpin conformation whereas the outer peptide is bent around the inner one, forming a nest-like structure. At high temperature the most common dimer conformation are NC-antiparallel and nested-antiparallel whereas at low temperatures CC-parallel, NN-antiparallel and CC-antiparallel dominate. At present, the precise nature, conformation and time evolution from Aβ monomers into intermediates are still unknown.

The next step was to choose the most representative MD simulation among the 26 replicas corresponding at different temperatures in order to study the geometric and energy interaction between both monomers. Thirumalai *et al.* rationalized that a suitable approximation is to choose a conformation that maximizes the interaction energy (sum of the van der Waals, electrostatic and desolvation energy) between both monomers [25]. To do so, the average interaction energy of the last 2 ns of each MD simulation was calculated using MMPBSA (Molecular Mechanics-Poisson Boltzann/Surface Area) method as implemented in AMBER9. Thirumalai’s procedure led us to 305 K MD simulation as the most preferable trajectory to be analyzed in depth. In line with our selection, several computational studies [26] on the structural diversity of Aβ_10–35_ oligomers observed a thermal dependency on the Aβ_10–35_ self-assembly: the maximum density of dimers is found between 280 K and 330 K whereas single monomers are predominant at high temperatures (>350 K) and tetramer units dominate at low temperatures (<290 K).

The structure at 305 K corresponds to the NC-parallel and well defined segment Leu17-Ala21 conformation, which the dimer interface is characterized by electrostatic interactions between the two chains, with the largest contribution from the salt bridge between N-terminus(A) and either Glu22(B) or Met35(B) and Asp23(A)-Lys16(B) (where A and B are the two monomers in the dimer). We conjecture that this U-shaped bend structure and the salt bridge formations may be critical in oligomerization. Then the 305 K MD simulation was prolonged 6ns up to 25 ns of total time length. Finally, the 6 ns at the beginning were rejected and the 19 ns at the end were sampled at 4 ps intervals which were subsequently used to analyze energy decomposition and other geometric variables.

### 2.4. MMPBSA decomposition of the binding free energy, ΔG_bind_, and binding energy, ΔE_bind_

The calculation and decomposition of binding free energy, ΔG_bind_, between monomer A and monomer B to form a dimer complex were evaluated using MMPBSA (Molecular Mechanics-Poisson Boltzmann Surface Area) method as implemented in AMBER9 [27,28]. MMPBSA has consistently been shown to be a good method for comparing binding energies of similar ligands as it is case [27,29,30]. MMPBSA computes the binding free energy by using a thermodynamic cycle that combines the molecular mechanical energies with the continuum solvent approaches. The binding free energy was calculated according to the equation [31]:

ΔG_bind_ = <G_C_> – <G_A_> – <G_B_> (1)

where C, A and B stand for complex, monomer A and monomer B for sake of simplification. The free energy of each term was estimated as a sum of the three terms:

<G> = <E_MM_> + <G_SOL_> – T<S> (2)

where E_MM_ is the molecular mechanics energy of the molecule expressed as the sum of the internal energy (bonds, angles and dihedrals) (E_int_), electrostatic energy (E_ele_) and van der waals term (E_vdw_):

<E_MM_> = <E_int_> + <E_ele_>+ <E_vdw_> (3)

<G_SOL_> accounts for the solvation energy which can divided into the polar and nonpolar part. The polar part accounts for the electrostatic contribution to solvation and is obtained by solving the linear Poisson Boltzmann equation in a continuum model of the solvent. On the other hand, the other part accounts for the nonpolar contribution to solvation and represents the cost of creation a cavity inside the solvent. This is related linearly to the solvent accessible surface area [32]. Notice that <G_SOL_> implicitly includes the entropy unlike <E_MM_>. Finally, configurational entropies were computed by diagonalization of the cartesian coordinate covariance matrix following the method described by Schlitter [33] and extensively tested in protein systems. The entropic contribution (-T<ΔS>) was calculated to be -0.842 and -0.844 kcal/mol at 305 K for monomer A and monomer B, respectively, whereas the term for the complex was estimated in -1.716 kcal/mol. Therefore, entropy term only reaches a marginal -0.03 kcal/mol correction to the ΔG_bind_. This finding is in the line with those that state that the entropy contribution will be small in systems with similar ligands [27,34].

After including all simplifications and all the energetic terms for both monomers and the complex equation 1 can be reorganisated and expressed as:

ΔG_bind_ = <ΔE_MM_> + <ΔG_sol_> (4)

where <ΔE_MM_> is simply the change in the internal energy and <ΔG_sol_> the change in the solvation profile between both monomers and the final complex. Binding free energy was calculated using 4750 snapshots sampled with ptraj program every 4 ps; these snapshots cover the last 19 ns of the 305 K trajectory.

To provide further insight into the changes that occur in the energetic profile of the interaction over the course of the trajectory, we plotted the components of the binding energy with respect to time. Notice that this energy will be called hereafter as binding energy (ΔE_bind_) and not binding free energy (ΔG_bind_) since it does not compute average values but just single decomposition in a conformation. Then equations (1), (2), (3) and (4) are transformed according to the new terminology as follows: (1) ΔE_bind_ = E_C_ – E_A_ – E_B_, (2) E = E_MM_ + E_SOL_, (3) E_MM_ = E_int_ + E_ele_ + E_vdw_, (4) ΔE_bind_ = ΔE_MM_ + ΔE_sol_, respectively. This methodology has been also used elsewhere [35]. All energy contributions have been (1) normalised by substracting the contributions mean and (2) smoothed with the moving average algorithm before plotting them altogether. If the mean is subtracted the new distribution, with mean equal to zero, allows us to visually compare the energy evolution of several contributions in one single plot while keeping the variability of each series.

### 2.5. Software orchestration: Kepler scientific workflow

On the one hand, the variety of software to be used in the analysis of the molecular dynamics trajectory and, on the other hand, the idea to develop an automated procedure for the general analysis of dimer formation encouraged us to explore new methodologies. The following processes had to be orchestrated: (1) molecular dynamics trajectory obtained with AMBER9, (2) RMSD and hydrogen bonds with the ptraj program, (3) MMPBSA calculation energies with the mmpbsa module within AMBER9 and finally (4) computational statistics with the R package [36]. The appropriate solution is offered by scientific workflows [37]. These are scientific procedures that combine data and processes into a configurable and structured set of steps with the aim at implementing automated computational solutions of a scientific problem. Kepler was the workflow environment chosen, a system for designing, executing, reusing, evolving, archiving and sharing scientific workflows [38].

Therefore, a unique Kepler workflow was designed to combine the four analyses in an orchestrated way. The first module builds a matrix with the decomposition of binding energy: 4750 snapshots as rows by 18 binding energy components (E_MM_, E_int_, E_ele_, E_vdw_, E_sol_ for the complex and both monomers and finally ΔE_bind_, ΔE_MM_ and ΔE_sol_ for the binding energy). The second module assembled the hydrogen bond matrix, a matrix of 4,750 snapshots by 420 hydrogen bonds that indicates presence (1) or absence (0) of such a bond. Finally, the third module got the dissimilarity RMSD matrix (4,750 by 4,750) computed by the ptraj program. All three matrices were intensively analysed by means of R software environment. Thus, the final workflow takes the AMBER trajectory as input and outputs a list of plots in an automatic procedure modulated by the parameters that user specifies. This Kepler’s workflow will be provided by the authors upon request.

## 3. Results and Discussion

### 3.1. Decomposition of the binding free energy, ΔG_bind_

MMPBSA stability calculations estimate the binding free energy (ΔG_bind_) between both monomers over the course of the 19 ns trajectory about -16 kcal/mol. The contributions of the molecular mechanics part (<ΔE_MM_>) and the solvation part (<ΔG_sol_>) are calculated to be -277.8 kcal/mol and 261.9 kcal/mol, respectively, see Table 1. Therefore, this reaction exemplifies a classical favorable reaction in solution where the increase of the stability produced by the formation of the dimer overcomes the cost of desolvation of both monomers. As explained in the introduction section, the minimization of the desolvation energy is expected to be primarily determined by the hydrophobic-hydrophobic interface contacts and the simultaneously localization of the hydrophilic parts in the outer surface of the dimer. On the other hand, the unfavorable change in energy associated with the burial of the polar residues in the interface does not influence dramatically the value of the desolvation energy as explained elsewhere [5,6].

**Table 1:** Decomposition of binding free energy, ΔG_bind_ in kcal/mol, according to equation 1 (ΔG_bind_ = <G_C_>− <G_A_> − <G_B_>) and equation 4 (ΔG_bind_ = <ΔE_MM_> + <ΔG_sol_>) ^1^.

*Equation (1)*	*ΔG_bind_*	*<G_C_>*	*<G_B_>*	*<G_A_> *
	-15.9 (10.9)	-904.7 (36.2)	-443.0 (22.2)	-445.8 (19.5)
*Equation (4)*	*ΔG_bind_*	*<ΔE_MM_>*	*<ΔG_sol_>*	
	-15.9 (10.9)	-277.8 (55.7)	261.9 (51.5)	

^(1)^ Standard deviation values in parenthesis.

Since we are not just interested in the values of ΔG_bind_ but the evolution of the constituents of binding free energy decomposition throughout the trajectory, the standard deviation of all energy components have also been included in Table 1. Such statistical information gives a proof of the intensity of the evolution of each monomer and the final dimer. It can be seen that monomer B experiments slightly higher energy variation than its counterpart.

### 3.2. Decomposition of the binding energy, ΔE_bind_, over the course of the trajectory

**Figure 1:**
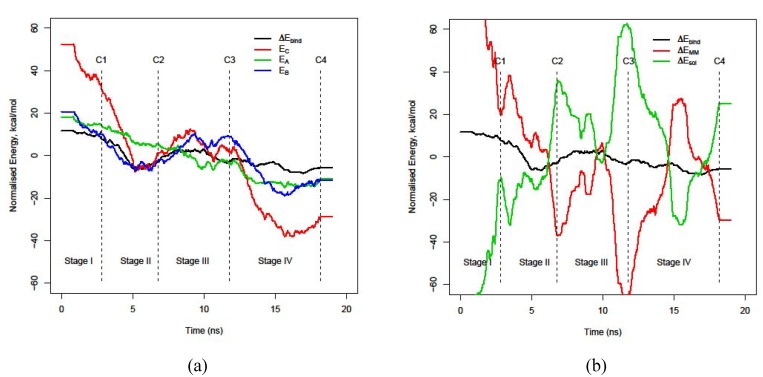
Evolution of ΔE_bind_ decomposition according to ΔE_bind_ = E_C_ − E_A_ – E_B_ and ΔE_bind_ = ΔE_MM_ + ΔE_sol_.

The plot of the decomposition of the binding energy, ΔE_bind_, (see Figure 1a) into its constituents (dimer, monomer A, monomer B) gives us with an overview of the general evolution of the energetics of the molecular interaction between both peptides. In this energy stability plot, four stages can be identified as a consequence of the different energy patterns of the dimer, monomer B and binding energy at specific trajectory regions. Notice that monomer A does not follow that pattern but rather a steadily stabilization along the trajectory. Stage I, which is called preparation stage, is characterized by an overall stabilization of all parts and it lasts approximately until 2.8 ns. In stage II, a local minimum of stability for the complex, monomer B and ΔE_bind_ is found around 5.0 ns. This pattern is observed again at the end of the trajectory at the last 6 ns (stage IV, called global stability), but with the difference of being a global instead of local minimum of ΔE_bind_. Local and global minimum points at this stability plots mean local stability, the opposite is valid for maximum points. Both stages are connected by an energy barrier: stage III. This stage, between 6.8ns and 11.7 ns, shows low stability for the complex, monomer B and the lowest binding energy between both monomers.

**Figure 2:**
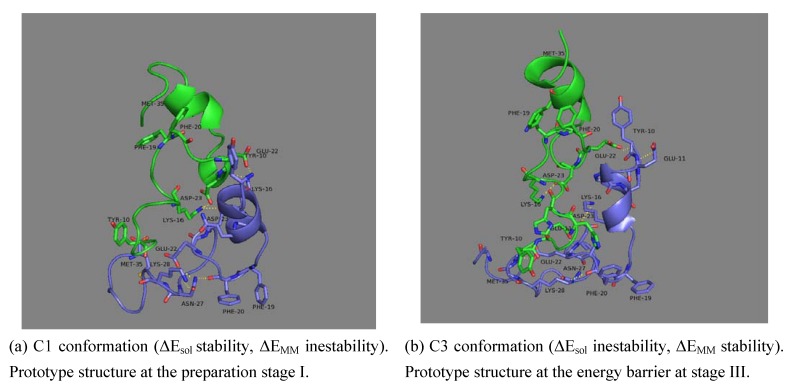
Geometric evolution of both monomers within the dimer structure.Monomer A (in blue) and monomer B (in green) at C1 (2.80 ns, between stage I and stage II) and at C3 (11.7 ns, at then end of stage III). The side-chains at the dimer interface are depicted explicitly.

In order to gain an insight into the factors that may drive such a process, the decomposition of binding energy into the molecular mechanics (ΔE_MM_) and solvation (ΔE_sol_) parts is also undertaken, see Figure 1b. It can be seen that the four stages are delimited by conformations of minimum MM energy and maximum solvation energy: 2.8, 6.8, 11.7 and 18.2 ns. This dichotomy of maximum solvation energy/minimum MM energy and vice versa is repeated along the trajectory and its fragile balance seems to determine the binding energy. These selected conformations, which are located in all figures by dotted lines and the numbering system C1, C2, C3 and C4, illustrate the most diverse kind of interactions found in the trajectory. Conformation C2 and C3 are depicted in Figure 2 as well as a description of the dominant effects that they represent. As Figure 1b shows, the local stability of ΔE_bind_ in stage II is produced by the drop of ΔE_MM_ inestability whereas ΔE_sol_ follows the opposite effect. On the other hand, the global stability of ΔE_bind_ in stage IV is due to a solvation effect: ΔE_sol_ yields energy values below the average value whereas ΔE_MM_ gets more unstable energy values. The energy barrier at stage III is caused by an increase of the ΔE_MM_ energy that cannot be compensated by the decrease of the desolvation energy.

**Figure 3:**
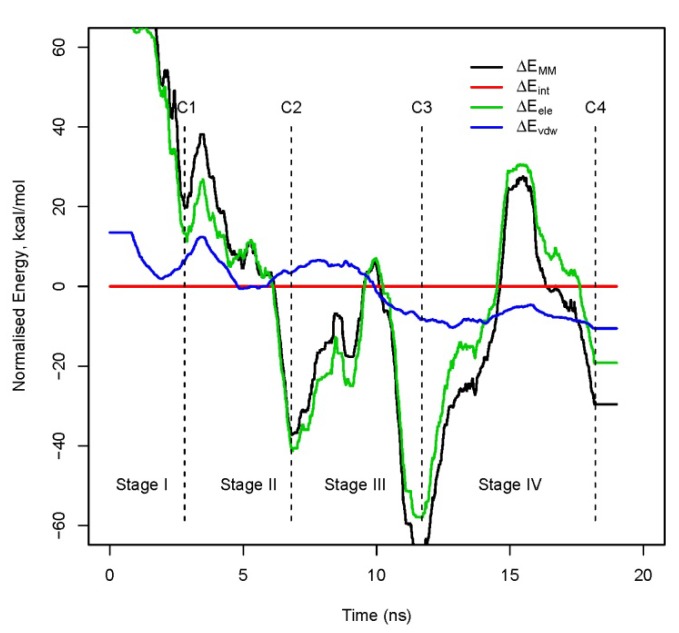
Evolution of ΔE_MM_ decomposition according to ΔE_MM_ = ΔE_int_ + ΔE_ele_+ ΔE_vdw_.

Finally, the decomposition of the molecular mechanics energy (ΔE_MM_) as a crucial part ΔE_bind_ into the into electrostatic (ΔE_ele_), van der Waals (ΔE_vdw_) and internal (ΔE_int_) can contribute to clarify which kind of interaction has changed the most between Stage II (local stability) and Stage IV (global stability). It should be notice first that the electrostatic term makes the prominent contribution to ΔE_MM_ and it is always present along the trajectory. Figure 3 determines that both electrostatic and van der Waals interactions increase their contribution to ΔE_MM_ from Stage II to IV, although the latter by steady stabilization and the former by more dramatic changes. Hydrophobic contacts at the interface (ΔE_vdw_) are preserved and even increased from the initial conformations to the lowest energy dimer structure at the end of the simulation. This is the sign of what has been called as the “hydrophobic collapse” and what could be called as the most significant energetic change observed in the trajectory. In contrast, the electrostatic interactions, which are stronger and more specific than the hydrophobic interactions, lead to a distribution that is more strongly peaked (ΔE_ele_) as it was already shown in references 26–30 but with a crucial presence along the trajectory.

These changes in the energy profile will be reflected in the geometric conformations of monomers and complex. Dimer conformations at minimum ΔE_MM_ between stages I/II (C1) and III/IV (C3) are displayed in Figure 2. These geometry representations highlight the ability of in-silico methods to uncover interactions that averaging methods such as NMR cannot. The geometric inspection reveals that the hydrophobic or van der Waals interactions occur frequently and simultaneously in stage III and IV unlike conformations in stage I and II. The hydrophobic core LVFFA, which displays a bend between Val24 and Lys28, is the responsible of the increase of hydrophobic interactions. Other observed hydrophobic events were characterized by the packing of the Phe19, Phe20, Val24 and Lys28. Example of hydrophilic interactions are the contact between Lys16(A/B) and either Glu22(A/B) or Asp23(A/B). Electrostatic interactions like the Glu22(A)-Lys16(B) and Asp23(A)-Lys16(B) are mutually exclusive with the Glu22(A)-Lys28(B) and Asp23(A)-Lys28(B), consistent with the flipping of the Lys28 side chain observed by Borreguero *et al.* [39] This intra-hydrogen bond is the most frequent within the monomers and is responsible for the internal stabilization of each monomer.

### 3.3. Energy stability of each monomer, E_A_ and E_B_

**Figure 4:**
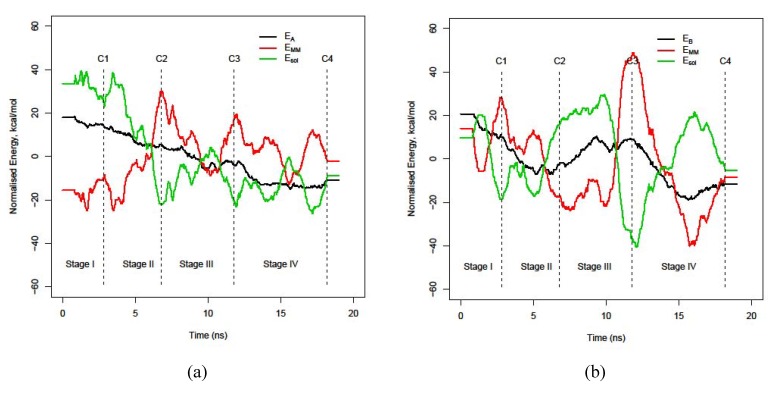
Evolution of the decomposition. (a) E_A_= E_MM,A_ + E_sol,A_ and (b) E_B_ = E_MM,A_ + E_sol,A_.

The decomposition of the energy for each monomer into molecular mechanics (E_MM_) and solvation energy (E_sol_), as in equation E = E_MM_ + E_sol_, completes the overall picture, see Figure 4. A visual inspection reveals that energy components of monomer B vary more fervently than those in monomer A, especially in the last three stages of the dynamic interaction. This corroborates also the finding in Figure 1 that whereas monomer A gets a steady stabilization, the energy profile for monomer B fluctuates backwards and forwards. Actually, both monomers adopt just two kinds of structures to interact and evolve towards the final stable dimer structure. The first type is characterized by a maximum stability in the solvation energy part and minimum stability in the molecular mechanics energy. This is a well-solvated structure. The second type of structure is simply the opposite balance: maximum stability in the molecular mechanics energy part whereas minimum in the solvation part. This represents a more compacted geometry for monomers. Both monomers exchange each type of conformation consecutively to achieve maximum stability for the overall dimer. In general, both monomers adopt the complementary type of structure of its counterpart except in the region of the energy barrier. The energy barrier around 10 ns in Figure 1a is caused by both monomers adopting a similar type of structure, which caused high energy instability. Notice also that each monomer stabilizes by means of a different mechanism, monomer B by solvation but monomer A by a molecular mechanics energy effect.

### 3.4. Root mean square deviation (RMSD) and hydrogen bond analysis

**Figure 5:**
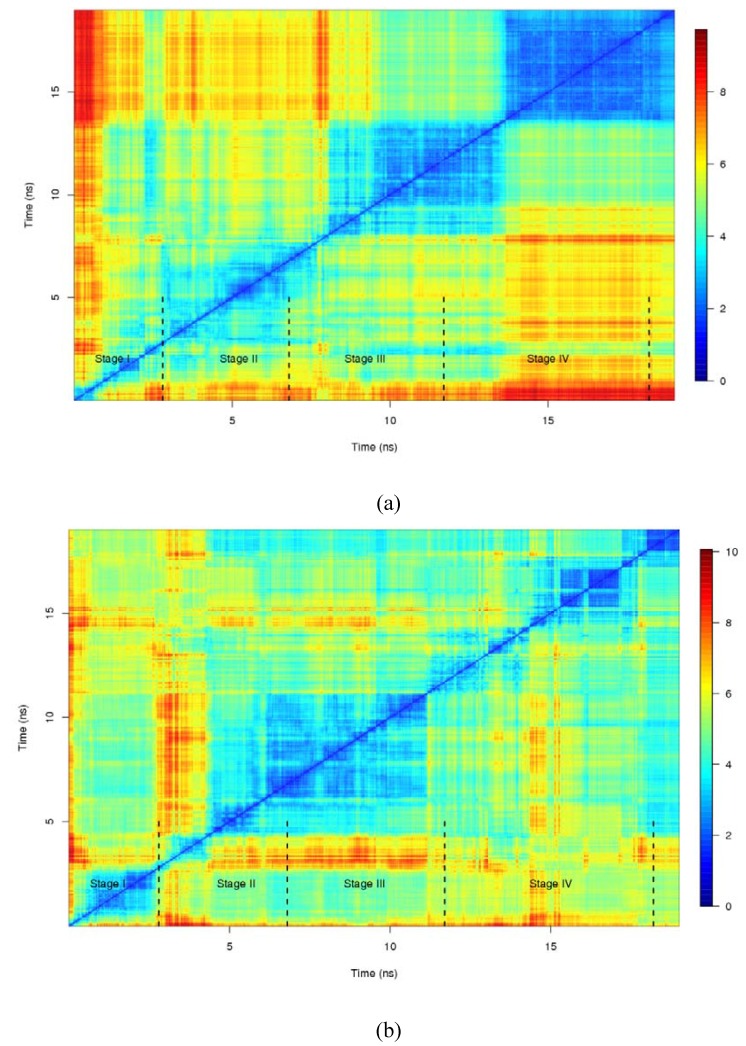
RMSD dissimilarity plot for (a) monomer A and (b) monomer B.

Figure 5 displays the RMSD dissimilarity matrix between the structures of monomer A and monomer B along the trajectory. Unlike the red dots, blue chunks represent structures geometrically similar and therefore they indicate the path that the monomer has adopted. Although RMSD is not as informative as binding energy decomposition, some complementary and corroborative information can be extracted from it.

First of all, the range of dissimilarity distances for monomer B (0–10) exceeds in one unit of RMSD that range for monomer A (0–9). This is in the line of what it has found in the previous section about the energy ranges. Moreover, unlike monomer B that moves geometrically backward and forward, monomer A follows a trajectory where final structure has nothing in relation with the starting point. However, as RMSD computes an average measure of changes it is not capable to capture the details found in the decomposition of the binding energies. See that thee four prototype structures form also four colored clusters in the RMSD plot. A visual inspection of the trajectory confirms also that the most part of the RMSD fluctuation comes from the N and C termini which suggest that there is a small contribution of those terminal regions of the peptide to the dimer association. Massi *et al.* [40] also reached this conclusion.

**Figure 6:**
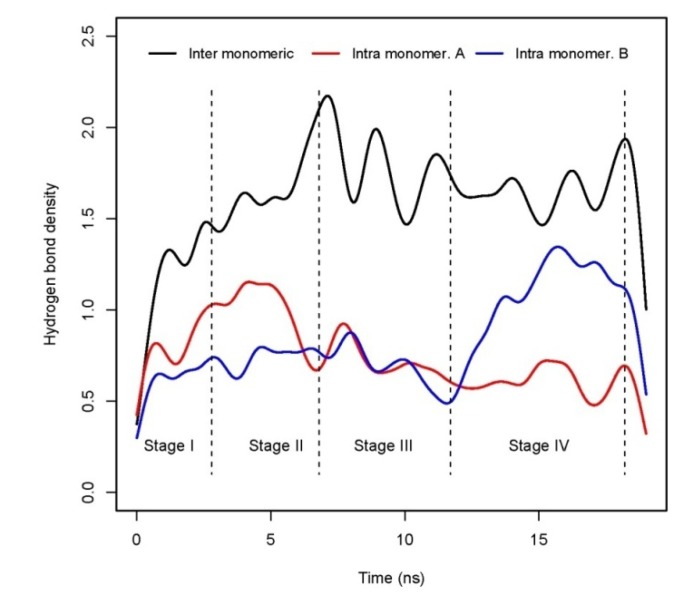
Evolution of hydrogen bond density for inter monomeric and intra monomeric types.

Finally, to get more support to the findings of binding energy decomposition, the hydrogen bonds density has been calculated along the trajectory. All of them have been grouped into three types: inter- hydrogen bonds between both monomers, intra-hydrogen bonds in monomer A and in monomer B. All hydrogen bonds, without an occupation threshold, are computed in the density plot in Figure 6.

This plot it is very illustrative of the overall dynamic process. Inter-hydrogen bond density is an indicator of the electrostatic interaction (ΔE_ele_) between both monomers. On the one hand, inter-hydrogen bonds that keep the complex bound attain a stable value around 1.5 from 3 ns up to the end, with the exception of the range between 6ns to 11 ns where pronounced peaks of presence and absence are found. This is in agreement with Figure 3, where ΔE_ele_ term keeps a constant value along the trajectory. The salt-bridge Glu22(A)-N-terminus(B) is the most frequent inter monomeric hydrogen bond, see Table 2. This hydrogen bond stabilizes the contact between N-terminus(A) in monomer A and the LVFFA hydrophobic core of monomer B. On another hand, intra hydrogen bond density profiles for both monomers track the changes in the electrostatic part (E_ele_) of the molecular mechanics energy (E_MM_). As seen in Table 2 and Figure 4, monomer A changes from a structure stabilized by E_MM_ energy (high presence of intra hydrogen bonds, thus high values of E_ele_) at the beginning of the interaction to an structure stabilized by solvation effects at the end of the trajectory (low presence of those hydrogen bonds, thus less contribution from E_MM_ term), the opposite effect is observed for the monomer B that gets stabilized by an electrostatic (E_ele_) energy effect corroborated by the high density of intra hydrogen bonds at the last part of the simulation.

**Table 2:** The five most frequent hydrogen bonds.

*No.*	*residue-residue*	*Type of hydrogen bond*	*Occupation, %*	*Comments*
1	Glu22(A)-N/terminus(B)	Inter monomeric	34.4	
2	Lys16(B)-Asp23(B)	Intra monomer B	23.5	Equivalent to Lys16(A)-Asp23(A)
3	Asp23(A)-Lys16(B)	Inter monomeric	23.4	
4	Lys16(A)-Asp23(A)	Intra monomer A	22.1	Equivalent to Lys16(B)-Asp23(B)
5	Lys16(A)-Asp23(B)	Inter monomeric	17.0	

### 3.5. Geometrical description of the final dimer conformation

**Figure 7:**
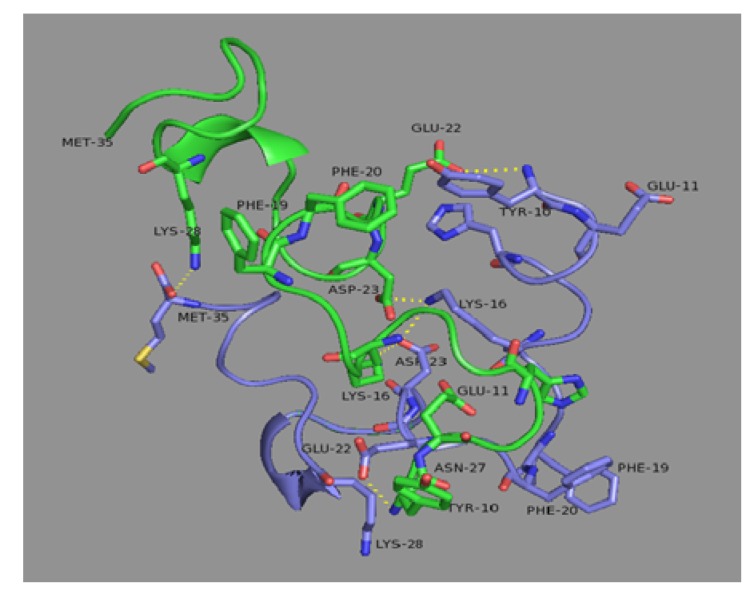
Final dimer complex conformation, C4 at 18.2 ns. The side-chains at the dimer interface are depicted explicitly. Monomer A is displayed in blue and monomer B in green.

The most stable dimer conformation corresponds to an anti-nested anti-parallel type, see Figure 7. As seen in the previous section, the large difference in RMSD values from the initial monomer conformation in stage I to the final conformations in stage IV indicates that the aggregation process has caused a large distortion. As RMSD and energy analysis pointed out, monomer B is more distorted than monomer A. However, in both monomers the geometric conformation of the hydrophobic core, LVFFA(17−21)(A) and LVFFA(17−21)(B), is conserved during the simulation. Another fact is the predominant random coil as a secondary structure with the minority presence of α-helix.

The contact map between residue side-chains (distance between their geometric centers less than 6.5 Å) was also computed. The salt bridge contacts that make the largest contribution are Lys16(A)-Asp23(B) and Asp23(A)-Lys16(B), seen also in Table 2. In the same region one can observe contacts between Ile32(A) and Leu17(B). The N-terminus(A)-LVFFA(B) contact is present but the predicted stability is almost entirely due to the favorable electrostatic interaction resulting from the salt-bridge Glu22(A)-N-terminus(B). Finally, the Met35(A)-Lys28(B) contact in which C-terminus(A) is involved with the β-turn(B) exemplifies an hydrophobic contact.

Due to the absence of any experimental structure of the Aβ_10−35_ dimer, it is difficult to identify the biological relevance of the final dimer conformation. However, solid-state NMR studies of Aβ fibrils have revealed a parallel in-register organization in β-sheets for both Aβ_10−35_ and Aβ_1−40_. Those structures raise the question of by what mechanism the native collapsed random coil structure of the Aβ_10−35_ monomer undergo conformational transition to a β-strand conformation that is characteristic of fibrils. Recent experimental studies have led to the conjecture that a transient α-helical phase is a necessary on-pathway intermediate connecting the monomeric peptide with the β-strand conformations of the fibrils.

## 4. Conclusions

Experimentalists have demonstrated that amyloid-beta (Aβ) peptides adopt a conformation mixture of random coil, α-helix and β-sheet in aqueous solution with the tendency to aggregation and fibrillization. Apparently, this conformation mixture and the conformational change toward fibrils make it extraordinarily difficult to design new inhibitors because Aβ peptides are basically nonstructural, and the intermediate conformation that could be the effective target is simply unknown. To circumvent this problem molecular, dynamics simulations were employed to study the conformational progression of Aβ dimers.

The MMPBSA decomposition of the binding energy into electrostatic, van der Waals and solvation contributions allow us to analyze in depth the energy profile of the interaction between both monomers and therefore determine the causes of the conformational evolution. MMPBSA decomposition proves that the electrostatic interaction between both monomers contribute predominantly and continuously to the binding energy along the simulation. The constant presence of inter monomeric hydrogen bonds supports this fact. Salt bridges between Lys16(A)-Asp23(B) and Lys16(B)-Asp23(A) and Glu22(A)-N-terminus turn out to be especially significant for the dimer stability. In line with this finding, studies of the effects of Asp23 kinetics have revealed also the importance of this residue in controlling fibrillogenesis [14].

Additionally, and not less important, at the last part of the simulation, only, the hydrophobic contribution calculated as van der Waals interactions gains importance. The hydrophobic contribution to the stabilization is exemplified by the structural stability of the hydrophobic central cluster LVFFA (residues 17 to 21). In other words, the hydrophobic central region tends to become exposed in the interchain between both monomers, thus forcing the hydrophilic residues to be located at the outside surface and consequently improving the solvation profile of the whole dimer. The increase of the solvation energy at the last part of the simulation has been also registered in our MMPBSA calculations. It is important to notice that this hydrophobic process happens without disturbing the crucial hydrophilic network of interactions between both monomers mentioned earlier.

Regarding the geometric conformations, the dimerization process involves a substantial structural reorganization of both C and N terminus regions though fewer changes in the central hydrophobic core are registered. Conversely, the β-strand propensity for Aβ_10−35_ dimer is negligible, with no residue having dihedral angles as typical for β-sheet. This finding suggests that β-sheets are not formed in the early stages of the disease when Aβ peptides are present as solvable entities.

The effect of the polymerization, formation of tetramers and octamers of Aβ peptides, on the energetic profile turns out to be the challenging next step in this research study. The number of monomers aggregated can influence profoundly the energy profile in a way to change the relative importance of electrostatic, van der Waals and solvation contributions to the binding free energy. The attention should be also focused on the design of new inhibitors of the aggregation and fibrillization processes. In that sense, compounds that destabilize and disaggregate Aβ oligomers will likely act by interfering with the formation of salt bridges formed by Asp23.
